# The production and secretion of tRNA-derived RNA fragments in the corn smut fungus *Ustilago maydis*

**DOI:** 10.3389/ffunb.2022.958798

**Published:** 2022-08-04

**Authors:** Rei Yoshimoto, Fumiko Ishida, Miyuki Yamaguchi, Shigeyuki Tanaka

**Affiliations:** Faculty of Agriculture, Setsunan University, Osaka, Japan

**Keywords:** *Ustilago maydis*, small RNA, tRNA, RNase T2, tRFs

## Abstract

The biogenesis of small non-coding RNAs is a molecular event that contributes to cellular functions. The basidiomycete fungus *Ustilago maydis* is a biotrophic pathogen parasitizing maize. A hallmark of its genome structure is an absence of RNAi machinery including Dicer and Argonaute proteins, which are responsible for the production of small RNAs in other organisms. However, it remains unclear whether *U. maydis* produces small RNAs during fungal growth. Here we found that *U. maydis* cells accumulate approximately 20-30 nucleotides of small RNA fragments during growth in the axenic culture condition. The RNA-seq analysis of these fragments identified that these small RNAs are originated from tRNAs and 5.8S ribosomal RNA. Interestingly, majority of their sequences are generated from tRNAs responsible for asparagine, glutamine and glycine, suggesting a bias of origin. The cleavage of tRNAs mainly occurs at the position near anticodon-stem-loop. We generated the deletion mutants of two genes *nuc1* and *nuc2* encoding RNase T2, which is a candidate enzyme that cleaves tRNAs. The deletion mutants of two genes largely fail to accumulate tRNA-derived RNA fragments. Nuc1 and tRNA are co-localized at the tip of budding cells and tRNA fragment could be detected in culture supernatant. Our results suggest that specific tRNAs would be cleaved during secretory processes and tRNA fragments might have extracellular functions.

## Introduction

Transcriptome analysis using high-throughput sequencers has revealed the presence of many noncoding RNAs (Mercer et al., 2009). Non-coding (nc) RNAs can be roughly classified into two major categories, small ncRNAs (< ~200 nt) and long ncRNAs (> ~200 nt). The smallest ncRNAs include the RNAs of 20–30 nt that are divided into small interfering RNA (siRNA) and microRNA (miRNA). In the 1990s, the phenomenon of “RNA silencing”, in which the expression of endogenous genes is suppressed by the introduction of transgene in plants and fungi, were reported (Napoli et al., 1990; Romano and Macino, 1992). Later this phenomenon was shown to be based on the production of short double-stranded RNA (dsRNA) and was named RNA interference (RNAi). RNAi is an evolutionary conserved phenomenon among eukaryotic species. Another category of small ncRNA of 20–30 nt include tRNA fragments (tRFs) (Shigematsu et al., 2014). tRFs are implicated in various cellular processes, such as apoptosis, gene silencing, translational inhibition, stress granule formation, cell growth inhibition, symbiosis, and more. However, the molecular functions of tRFs remain largely unknown, except that tRFs are further processed into siRNA and involved in gene-silencing (Maute et al., 2013; Kumar et al., 2014; Kuscu et al., 2018; Ren et al., 2019).

The basidiomycete fungus *Ustilago maydis* is a biotrophic pathogen causing smut disease in maize and able to infect all aerial part of maize plant (Banuett, 1995; Brefort et al., 2009; Zuo et al., 2019). Characteristic disease symptom induced by *U. maydis* is tumor formation on the infected maize tissues. The genome of *U. maydis* encodes many genes for effector proteins that are secreted during plant colonization and contribute to virulence (Kämper et al., 2006; Lanver et al., 2017). The genes encoding effector proteins in *U. maydis* are specifically upregulated during host colonization and are expressed during the course of fungal development on and inside the plant tissue (Lanver et al., 2018). Another hallmark of the genome structure of *U. maydis* is an absence of genes responsible for RNAi machinery (Kämper et al., 2006; Laurie et al., 2012; Billmyre et al., 2013). The genome of *U. maydis* lacks the genes encoding RNA-dependent RNA polymerase, Dicer and Argonaut proteins due to clean excision events, while the close relatives including *U. hordei* and *Sporisorium reilianum* maintain these genes (Laurie et al., 2012). Similarly, *Saccharomyces cerevisiae* also lacks RNAi machinery, which has been explained by the incompatibility between RNAi and double-stranded RNA killer viruses (Drinnenberg et al., 2011), suggesting that it could be also advantageous for *U. maydis* to possess toxin-producing killer virus in its lifestyle. To visualize whether *U. maydis* actually does not produce small RNAs from double-stranded RNA *via* Dicer, Laurie et al. attempted to express double-stranded RNA artificially in *U. maydis* and found that short interfering RNAs could not be detected (Laurie et al., 2008). Their results propose the fact that *U. maydis* does not produce Dicer-dependent small RNAs. On the other hand, it has been shown that *U. maydis* produces hundreds of natural antisense transcripts (NATs) as ncRNAs that corresponds to open reading frames (Ho et al., 2007; Donaldson and Saville, 2013). Altered expression of NAT in *U. maydis* has been also shown to result in virulence reduction in maize (Donaldson and Saville, 2013), suggesting that the production of ncRNAs in *U. maydis* would contribute to the regulation of gene expression.

In addition to the precedent works, we found the accumulation of small RNAs in sporidia and filamentous cells of *U. maydis* in this study. Majority of these small RNAs were derived from 5’ end of specific tRNAs and 3’ end of 5.8S ribosomal RNA. These RNA fragments were generated by cleavage of stem loop, which was achieved by two RNase T2 enzymes in *U. maydis*. RNase T2 protein and tRNA were co-localized at the tip of budding cells, and tRF derived from tRNA-Gly could be detected in culture supernatant. Our work proposes the possible extracellular function of tRFs in *U. maydis*.

## Material and methods

### Growth conditions and virulence assays

The seeds of *Zea mays* cv Golden Bantam was obtained from Bingenheimer Saatgut AG (Echzell-Bingenheim, Germany) and propagated at the agricultural field of Setsunan University in Japan. To assess the virulence of *U. maydis*, maize plants were grown in a temperature-controlled phytochamber (16 h light (420 μmol m^-2^ s^-1^), 8 h dark, 26°C, 40% RH). The solopathogenic strain SG200 of *U. maydis* has been described previously (Kämper et al., 2006). For virulence assay, *U. maydis* strains were grown in YEPSL (0.4% yeast extract, 0.4% peptone, 2% sucrose) on a rotary shaker (200 rpm) at 28°C for overnight. Cells were inoculated to fresh YEPSL as OD_600_ is 0.2 to prepare subculture. Once OD_600_ reached to 1.0, cell pellet was resuspended in water and injected into the stem of 7-days-old maize seedlings with a syringe as described previously (Kämper et al., 2006). Disease symptoms were scored at 12 days post infection using a previously developed scoring scheme (Kämper et al., 2006). The percentage of each disease categories were calculated in each replicates and were presented as box plots. The significant differences of each disease symptoms were statistically assessed by one-way ANOVA test.

### Plasmid construction and generation of *U. maydis* mutants

The Gibson Assembly Master Mix (New England Biolabs, Ipswich, MA, U.S.A.) was used for plasmid construction according to the manufacturer’s protocol. All primers used for DNA amplification are listed in **Supplementary Table S1**. The fungal strains used in this study are listed in **Supplementary Table S2**.

To generate deletion mutants of *nuc1* in *U. maydis*, the deletion construct pKO_HygR_UMAG_02611 was generated as follows. The left and right border regions (1000 bp each) of *nuc1* were amplified by PCR with primers UMAG_02611_LB-FW and UMAG_02611_LB-RV and primers UMAG_02611_RB-FW and UMAG_02611_RB-RV, respectively. Hygromycin resistance marker cassette was amplified with primers UMAG_02611_Hyg-FW and UMAG_02611_Hyg-RV from plasmid pBS-Hyg (Molina and Kahmann, 2007). These three fragments were assembled using the Gibson Assembly Master Mix into pBlueScript that has been linearized by *Eco*RI–*Bam*HI. A 4 kb fragment containing the hygromycin resistance marker cassette flanked by the left and right border regions of *nuc1* was excised from pKO_HygR_UMAG_02611 by *Ssp*I, and used for transformation of *U. maydis* SG200. Gene replacement mutants were identified by PCR and Southern blot analysis.

To generate double deletion mutants of *nuc1* and *nuc2* in *U. maydis*, the deletion construct pKO_GenR_UMAG_03023 was generated as follows. The left and right border regions (1000 bp each) of *nuc2* were amplified by PCR with primers UMAG_03023_LB-FW and UMAG_03023_LB-RV and primers UMAG_03023_RB-FW and UMAG_03023_RB-RV, respectively. Geneticin resistance marker cassette was amplified with primers UMAG_03023_GenR-FW and UMAG_03023_GenR-RV from plasmid pKO_GenR_SrTin2 (Tanaka et al., 2019). These three fragments were assembled using the Gibson Assembly Master Mix into pBlueScript that has been linearized by *Eco*RI–*Bam*HI. A 4 kb fragment containing the geneticin resistance marker cassette flanked by the left and right border regions of *nuc2* was excised from pKO_GenR_UMAG_03023 by *Kpn*I-*Spe*I, and used for transformation of *U. maydis* SG200Δ*nuc1*. Gene replacement mutants were identified by PCR and Southern blot analysis.

To generate a strain expressing Nuc1-HA protein, the construct p123_UMAG_02611-HA was generated. Genomic DNA from SG200 containing promoter and open reading frame of *nuc1* was amplified by PCR with primers UMAG_02611_CP-FW2 and UMAG_02611_CP-RV. The amplified fragment was introduced into the p123_Sta1_HA (Tanaka et al., 2020) that has been linearized by *Nde*I-*Bst*EII. Prior to transformation, plasmid was linearized with *Ssp*I. Transformants were screened by PCR and Southern blot analysis. Strains that the plasmid was inserted in the *ip* locus (Loubradou et al., 2001) of SG200Δ*nuc1* were used for further experiments.

### RNA extraction, denaturing PAGE and Northern blot analysis

Total RNA was extracted from 4 ml overnight culture of sporidia or filamentous cells of *U. maydis* AB33 or SG200 with TRIzol reagent (Thermo Fisher Scientific, Waltham, MA U.S.A.) following manufacturer’s protocol. Filamentous cells of AB33 were induced in NM-liquid medium (0.3% KNO_3_, 6.25% salt solution (Holliday, 1974), pH 7.0) containing 2% glucose. Fungal cells were vortexed in 2 ml tube containing 100 μl glass beads and 500 μl TRIzol for 10 min. After centrifugation, supernatant was used for RNA precipitation at -80°C overnight. Total RNA concentration was determined by Qubit RNA BR Assay Kit (Thermo Fisher Scientific). RNAs in culture supernatant were collected as follows. Overnight culture of *U. maydis* strains was inoculated 4 ml YEPSL (OD_600_ = 0.2). After 6 hrs incubation, culture supernatant was collected after centrifugation. RNA isolation from culture supernatant was performed with TRIzol LS reagent (Thermo Fisher Scientific) following manufacturer’s protocol.

The RNA samples were dissolved in 50% formamide and separated by 15% denaturing polyacrylamide gel electrophoresis (PAGE) containing 8M urea. Electrophoresis was performed at constant voltage 180 V for approximately 150 min. RNA fragments were visualized by staining gel with GelRed (Takara Bio, Shiga, Japan). Northern blot analysis was performed according to a standard protocol using DIG-labeled DNA probe for tRNA-Gly (5´-DIG-ACGTCCCGTGATACCACTACACTACCAATGC-3´), which was synthesized by the manufacturer Fasmac (Kanagawa, Japan). The signal was detected by Amersham ImageQuant 800 (Cytiva, Marlborough, MA, U.S.A.).

### Small RNA-seq analysis and bioinformatics

Using small RNAs from filamentous and sporidia cells of *U. maydis* AB33, small RNA-seq library was prepared using NEXTFLEX Small RNA-Seq Kit v3 (Perkin Elmer, MA, U.S.A.) according to the manufacturer’s instruction. The library was sequenced on the MiSeq (Illumina, CA, U.S.A.) platform. Sequence reads were deposited on NCBI Sequence Read Archive (SRA) under accession number PRJNA843565. Sequence quality check and identification of overrepresented sequence reads were performed using fastqc (http://www.bioinformatics.babraham.ac.uk/projects/fastqc/) and blast (https://blast.ncbi.nlm.nih.gov/Blast.cgi). Predicted genomic and mature tRNA sequences (based on *Ustilago maydis* 521 genome) were downloaded from GtRNAdb (http://gtrnadb.ucsc.edu/GtRNAdb2/index.html) and were aligned to the genome. The sequence reads were mapped to the genome using Bowtie (Langmead et al., 2009) with -n 0 option and the obtained bam file were used to count the sequence reads using bedtools (v2.30.0). Boxplot was depicted using R (https://cran.r-project.org) and ggplot2 (http://www.bioconductor.org). Sequence reads and statistics are described in **Supplementary Table S3**.

### Western blot analysis

Hemagglutinin (HA)-tagged Nuc1 protein was detected as previously described (Tanaka et al., 2014; Tanaka et al., 2019). Sporidia cells were incubated until OD_600_ reaches to 1.0 in 4 ml YEPSL medium. After centrifugation, the cell pellet was used for the extraction of total proteins. Culture supernatant was filtered with 0.2 μm filter to remove residual sporidia cells. Secreted Nuc1-HA protein was harvested by TCA precipitation from culture supernatant as previously described (Tanaka et al., 2014). Proteins were separated by 12% sodium dodecyl sulfate (SDS)–polyacrylamide gel electrophoresis. A rabbit polyclonal anti-HA antibody (H6908; Sigma-Aldrich, St. Louis, MO, U.S.A.) or a mouse monoclonal anti-tubulin antibody (T9026; Sigma-Aldrich) were used as the primary antibody at 1: 10000 dilution to detect Nuc1-HA or tubulin protein, respectively. Anti-rabbit immunoglobulin G (IgG) horseradish peroxidase-linked antibody (Cell Signaling Technology, Danvers, MA, U.S.A.) or anti-mouse IgG horseradish peroxidase-linked antibody (Cell Signaling Technology) were used as a secondary antibody at 1: 10000 dilution. To detect signals, Amersham ECL select (Cytiva) was used as substrate for horseradish peroxidase and the signal was detected by Amersham ImageQuant 800 (Cytiva).

### Immunocytochemistry and *in situ* hybridization

To visualize the localization of Nuc1-HA protein and tRNA-Gly in the fungal cells, immunocytochemistry and *in situ* hybridization were simultaneously performed. Sporidia cells of *U. maydis* strain expressing Nuc1-HA were fixed with 3% formaldehyde in phosphate-buffered saline (PBS). To permeabilize, cells were incubated with 0.1% Triton-X100 for 10 min. The cells were hybridized with DIG-labeled DNA probe for tRNA-Gly at 42°C overnight. After washing, cells were incubated with rabbit anti-HA antibody (H6908; Sigma-Aldrich; 1: 500 dilution) and mouse anti-DIG antibody (ab420; Abcam, Cambridge, UK; 1: 500 dilution) in TBS-T containing 5% skim milk for 1 h at room temperature. After washing, cells were incubated in TBS-T containing goat anti-Rabbit IgG cross-adsorbed secondary antibody conjugated with Alexa Fluor 488 (R37116; Thermo Fisher Scientific) and goat anti-mouse IgG Cy3 conjugate (AP181C; Merck, Darmstadt, Germany; 1: 500 dilution) for 1 h at room temperature. DAPI staining was performed at final concentration 1 μg/ml for 5 min at room temperature. After washing with TBS-T, the samples were analyzed using a Zeiss Axio Observer 7 microscope equipped with Colibri 7 under filter set 38HE for Alexa 488, 45 for Cy3 and 96HE for DAPI fluorescence (Carl Zeiss, Oberkochen, Germany).

## Results

### Identification of small RNAs from *U. maydis* cells grown in axenic culture

**Figure 1:**
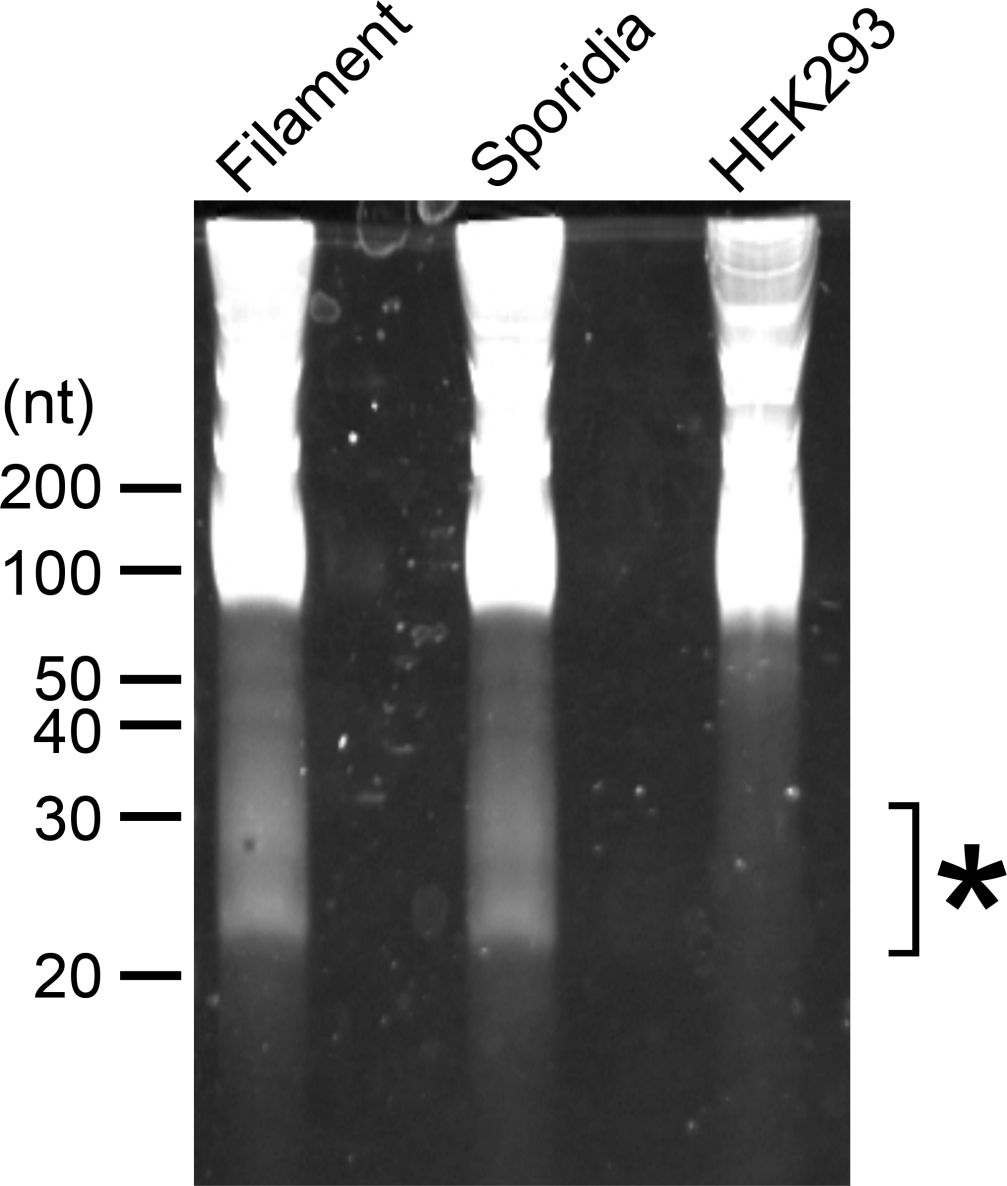
Electrophoresis of total RNAs extracted from *U. maydis*. RNA samples extracted from filamentous and sporidia cells of *U. maydis* AB33 strain were loaded on 15% denaturing PAGE containing 8 M Urea. Filamentous cells of AB33 were induced in NM-liquid medium and sporidia cells of AB33 were incubated in YEPSL. Total RNA from human HEK293 cells was loaded as negative control. Asterisk indicates the region used for the extraction of small RNA fragments from filamentous and sporidia cells and subsequent RNA-seq analysis.

**Figure 2:**
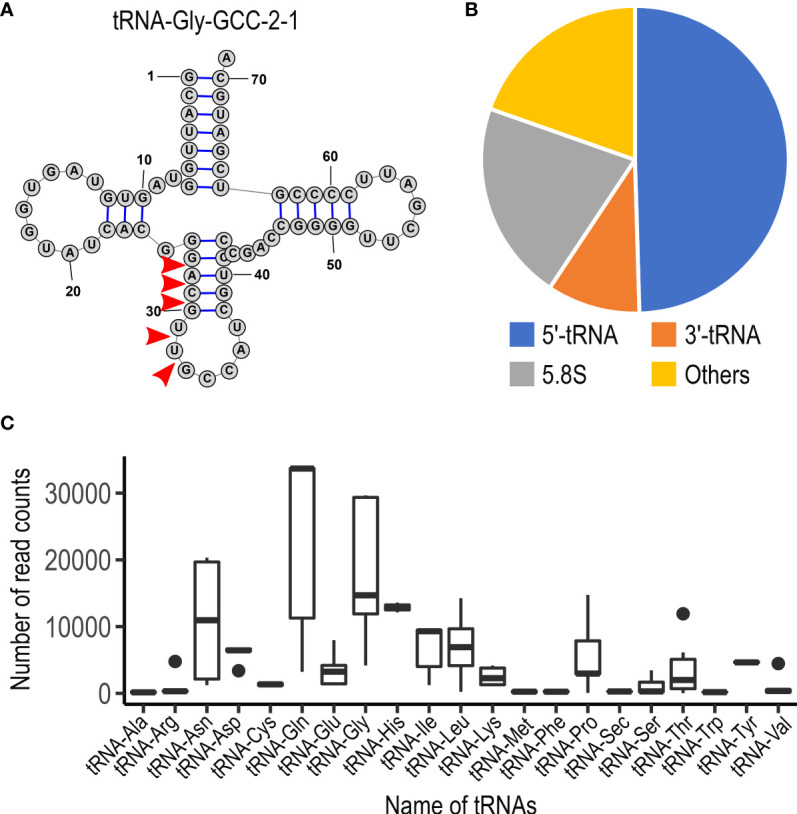
Characteristics of small non-coding RNAs in *U. maydis*. **(A)** Schematic picture of the structure for tRNA-Gly (GCC-2-1) in *U. maydis*. Red arrow heads indicate the putative cleavage sites, which are predicted from the sequence reads obtained by RNA-seq analysis. The numbers indicate the position of nucleotides from 5’ end of tRNA-Gly. **(B)** Pie chart showing the proportion of the type of small non-coding RNAs in *U. maydis*. **(C)** Box plot showing the number of sequence read counts derived from respective tRNAs. Dots represents outside values in each box plots.

To examine whether *U. maydis* cells accumulate small RNAs, we extracted total RNA from sporidia cells and filamentous cells of *U. maydis* AB33 strain. Approximately 30 μg total RNAs as well as human HEK293 total RNAs (30 μg) were separated on 15% denaturing PAGE gel containing 8M urea and we attempted to visualize small RNA fragments. Although HEK 293 cells are demonstrated to contain miRNAs, the amount of small RNA in human cells are very less amount and generally not visible on gel after electrophoresis. At the length between 20 and 30 nucleotides, we could observe several discrete bands in both sporidia and filamentous cells of *U. maydis* (**Figure 1**). This suggested that *U. maydis* accumulates small RNA fragments in fungal cells. Next, we extracted these small RNA fragments of filamentous and sporidia cells from gel and analyzed by RNA-seq using Illumina MiSeq to identify the respective sequences. Sequences obtained were mapped to the genome of *U. maydis* and overrepresented sequences (> 0.1%) were listed in **Supplementary Table S3**. The results revealed that these RNA fragments were mainly derived from tRNAs and 5.8S ribosomal RNA of *U. maydis* (**Supplementary Table S3**). The sequences clearly suggested that tRNAs were likely cleaved at the position near anti-codon stem loop (**Figure 2A**; **Supplementary Figure S1**). For 5.8S ribosomal RNA, the cleavage was likely occurred at the position between 125 and 130 nucleotides, which corresponds to the tip of stem loop (**Supplementary Figure S2**). Furthermore, approximately 50% of small RNA fragments were derived from 5’ end of tRFs rather than 3’ end (**Figure 2B**). In case of 5.8S ribosomal RNA, 3’ end of the fragments was detected (**Supplementary Figure S2**; **Supplementary Table S3**). These results indicate that the majority of small RNAs accumulated in fungal cells was 5’-tRFs. Interestingly, tRFs found in fungal cells were mainly from tRNA responsible for asparagine (tRNA-Asn), glutamine (tRNA-Gln) and glycine (tRNA-Gly) (**Figure 2C**). This suggests that the cleavage occurs at specific tRNAs rather than random degradation of total tRNAs.

### Two RNase T2 genes in *U. maydis* are responsible for the production of tRFs

**Figure 3:**
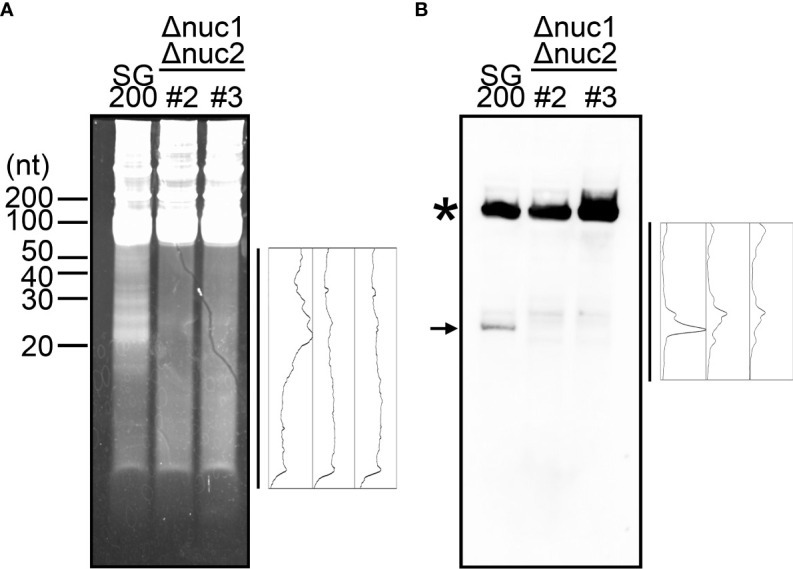
Visualization of small non-coding RNAs in the double deletion mutants of *nuc1* and *nuc2* in *U. maydis*. **(A)** Electrophoresis of total RNAs extracted from SG200 and SG200Δ*nuc1*Δ*nuc2* mutants (designated as #2 and #3). Densitometry of small RNA bands in an indicated area was shown at the right of panel. **(B)** Northern blot analysis for the detection of tRNA-Gly fragment in SG200 and SG200Δ*nuc1*Δ*nuc2* mutants. Asterisk indicates the full-length tRNA-Gly. Arrow indicates a major 5’-tRNA fragment generated from full-length tRNA-Gly. Densitometry of tRNA fragment signals in an indicated area was shown at the right of panel.

While Dicer and Ago proteins are generally responsible for the cleavage of tRNAs at the position near anticodon-stem-loop in many other organisms (Xie et al., 2020), it has been revealed that RNase T2 is responsible for it in the plant *Arabidopsis thaliana* and the yeast *S. cerevisiae* (Thompson and Parker, 2009a; Megel et al., 2019). The genome of *U. maydis* carries two genes encoding RNase T2, which has been designated as *nuc1* (*UMAG_02611*) and *nuc2* (*UMAG_03023*) in other work (Mukherjee et al., 2020). The *nuc1* gene is specifically upregulated during plant colonization according to the previous data from time-resolved transcriptome analysis, while *nuc2* is relatively weak expression through all time points (**Supplementary Figure S3**) (Lanver et al., 2018; Mukherjee et al., 2020). To examine whether *nuc1* is responsible for the production of tRFs, we firstly generated the deletion mutants of *nuc1* in the solopathogenic strain SG200 of *U. maydis*. Total RNA was extracted from sporidia cells of SG200Δ*nuc1* and loaded on the Urea PAGE to check the accumulation of small RNAs. However, the deletion strains accumulated small RNAs similar to SG200 (data not shown). To test the possibility that the second RNase T2 gene *nuc2* also contributes to the production of tRFs, we generated the double knockout mutants for *nuc1* and *nuc2*. Total RNA was extracted from sporidia cells of SG200Δ*nuc1*Δ*nuc2* and loaded on the Urea PAGE. Here we could largely not detect the accumulation of small RNAs in the double deletion mutants, although SG200 accumulates small RNAs (**Figure 3A**). To confirm whether tRFs are actually not generated, we performed Northern blot analysis using a probe that detects tRNA-Gly (GCC-2-1). In SG200, the strong signal appeared at upper position, which indicates full-length tRNA, and the major signal also appeared at lower position, which indicates tRF derived from tRNA-Gly (**Figure 3B**). In contrast, SG200Δ*nuc1*Δ*nuc2* mutants did not show the major signal appeared at lower position in SG200, while full-length tRNA signals were detectable at upper position (**Figure 3B**). Taken together, these results indicate that both two RNase T2 are mainly responsible for the production of tRFs in *U. maydis*.

### Two RNase T2 genes do not contribute to fungal virulence of *U. maydis*

**Figure 4:**
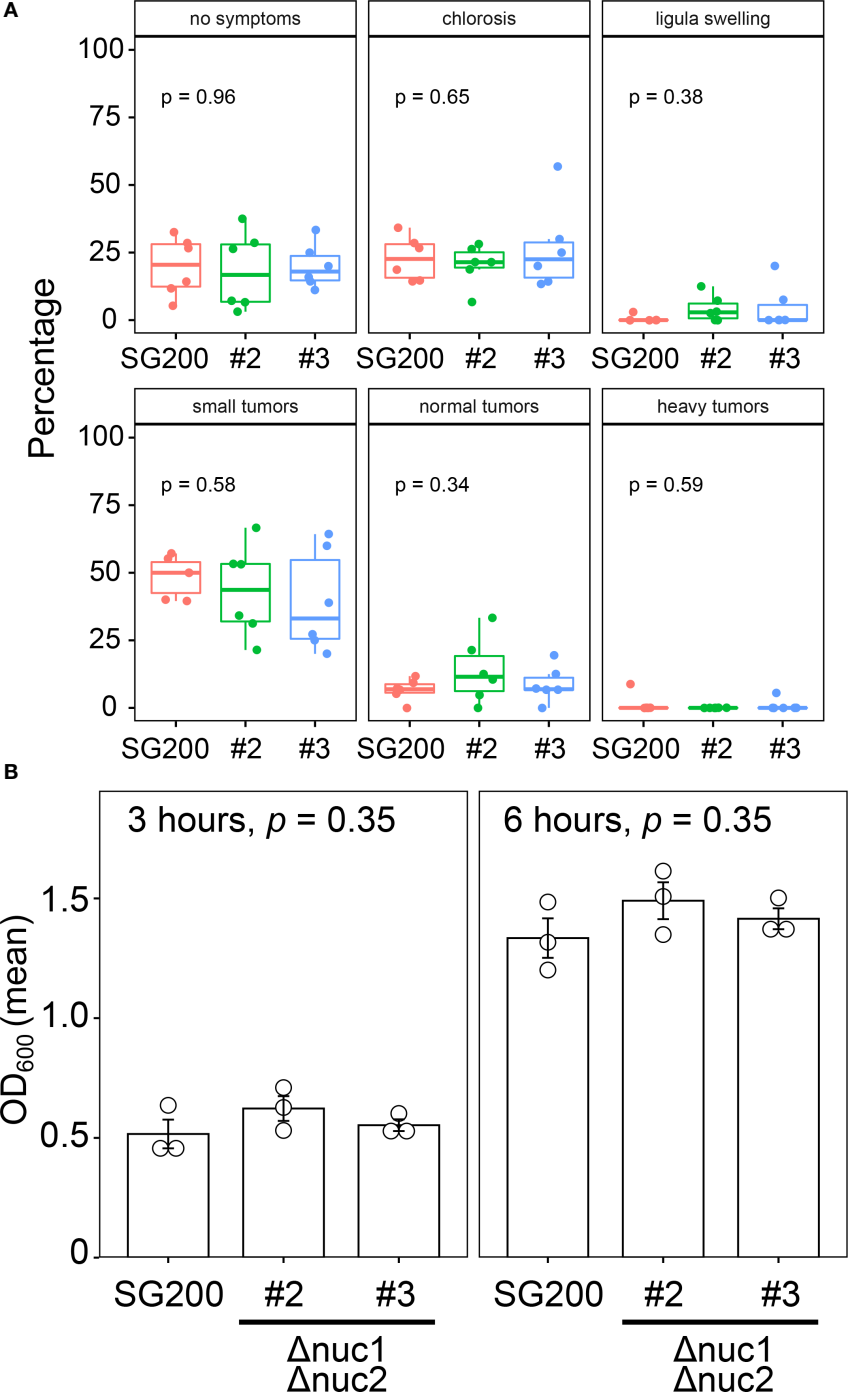
Virulence and growth ability of SG200Δ*nuc1*Δ*nuc2* mutants in *U. maydis*. **(A)** Pathogenicity assay of the double deletion mutants of *nuc1* and *nuc2* in *U. maydis*. Disease symptoms were quantified based on six biological replicates. The percentage of plants placed in a certain disease category is calculated in each replicate and represented as box plots. One-way ANOVA test was applied to detect significant difference of disease symptom among SG200 and SG200Δ*nuc1*Δ*nuc2* mutants. *p*-value is indicated in each disease category. **(B)** Growth rate of SG200Δ*nuc1*Δ*nuc2* mutants in *U. maydis*. Suspension of sporidia cells (OD_600_ = 0.2) were cultured in YEPSL liquid medium. Optical density (OD_600_) was measured after 3 hours and 6 hours cultivation, respectively. Three biological replicates (depicted as open circles) were prepared to determine the mean of optical density and standard errors. One-way ANOVA test was used to apply statistical analysis and *p*-values were indicated in respective panels of bar graph.

To examine the contribution of RNase T2 to fungal virulence in *U. maydis*, we inoculated the double deletion mutants of *nuc1* and *nuc2* genes to host plant maize. Here we tested two independent deletion mutants (designated as #2 and #3). At 12 days post inoculation, we scored the disease severity according to the schemes that has been developed in the previous works (Kämper et al., 2006). The inoculation assay was performed independently by six times and the percentage of disease symptoms in each replicates was represented as box plots in **Figure 4A**. To detect the significant differences of fungal virulence between SG200 and SG200Δ*nuc1*Δ*nuc2* mutants, we performed one-way ANOVA test. However, the statistically significant differences could not be detected in each disease categories between SG200 and SG200Δ*nuc1*Δ*nuc2* mutants (**Figure 4A**). This indicates that *nuc1* and *nuc2* genes are not important for virulence of *U. maydis*. To examine whether the double deletion of *nuc1* and *nuc2* affects fungal growth, we cultured the double deletion mutants in axenic culture and compared the growth rate compared to the strain SG200 of *U. maydis*. However, we could not detect statistically significant differences of fungal growth at neither 3 hours nor 6 hours post inoculation (**Figure 4B**). Taken together, the deletion of *nuc1* and *nuc2* genes are unlikely to impact on plant colonization and fungal growth.

### Cellular localization of Nuc1 and tRNA-Gly in *U. maydis* cells

**Figure 5:**
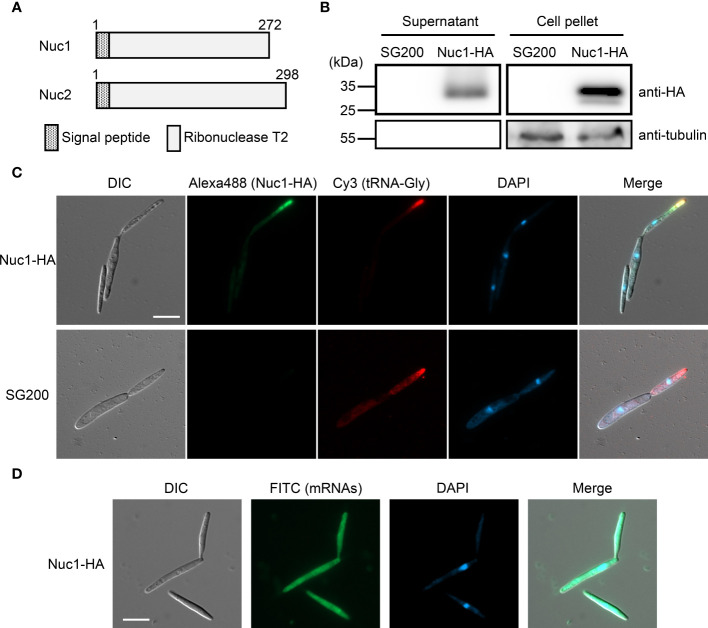
Cellular localization of Nuc1-HA protein in sporidia cells of *U. maydis*. **(A)** Schematic picture of Nuc1 and Nuc2 protein structures. Both proteins carry signal peptide at N-terminus. The entire protein sequence encodes ribonuclease T2 domain. **(B)** Western blot analysis of the strain expressing Nuc1-HA. Total proteins and secreted proteins from SG200 and SG200Δ*nuc1*-*nuc1-HA* were separated in 12% SDS-PAGE. Nuc1-HA protein was detected with anti-HA antibody and tubulin was detected with anti-tubulin antibody. **(C)** Immunocytochemistry and *in situ* hybridization for the detection of Nuc1-HA and tRNA-Gly in sporidia cells. Nuc1-HA localization was visualized by Alexa Fluor 488. tRNA-Gly localization was visualized by Cy3. Fungal nuclei were visualized by DAPI staining. Bar = 10 μm. **(D)** *In situ* hybridization for the detection of mRNAs in sporidia cells. mRNAs localization was visualized by FITC. Fungal nuclei were visualized by DAPI staining. Bar = 10 μm.

Both RNase T2 proteins Nuc1 and Nuc2 in *U. maydis* are predicted to carry a signal peptide at N-terminus (**Figure 5A**), indicating that the proteins are secreted to extracellular space *via* canonical endoplasmic reticulum (ER) to Golgi route. To confirm the protein secretion, we generated *U. maydis* strain expressing Hemagglutinin (HA)-tagged Nuc1 at C-terminus, and performed western blot analysis using HA antibody with proteins extracted from culture supernatant and cell pellet. In both supernatant and cell pellet, Nuc1-HA could be detected as an expected size (30.7 kDa) (**Figure 5B**). We also performed western blot using tubulin antibody and could detect tubulin only in cell pellet, suggesting the absence of cell lysis (**Figure 5B**). To understand where RNase T2 protein functions in fungal cells for the cleavage of tRNAs, we performed immunocytochemistry for Nuc1-HA and *in situ* hybridization for tRNA-Gly simultaneously. In the cells of the strain expressing Nuc1-HA, the strong fluorescent signals were mainly detected at the tip of budding daughter cells and visible as speckles (**Figure 5C**; **Supplementary Figure 4**), suggesting the localization in secretory vesicles. Interestingly, tRNA-Gly signals were also detectable at the tip of budding cells, indicating the co-localization with Nuc1-HA (**Figure 5C**; **Supplementary Figure 4**). In the control strain SG200, Nuc1-HA signals could not be detected but tRNA-Gly signals were detectable at the tip of budding cells similar with the strain expressing Nuc1-HA (**Figure 5C**). To make sure that the localization pattern of tRNA-Gly is specific, we also performed *in situ* hybridization for mRNAs using oligo dT probe in the strain expressing Nuc1-HA. The mRNA signals were evenly detected inside sporidia cells (**Figure 5D**), indicating the localization to the tip of budding cells is specific to tRNA-Gly. Taken together, these results suggest that Nuc1-HA would co-localize with tRNAs through secretory pathway.

**Figure 6:**
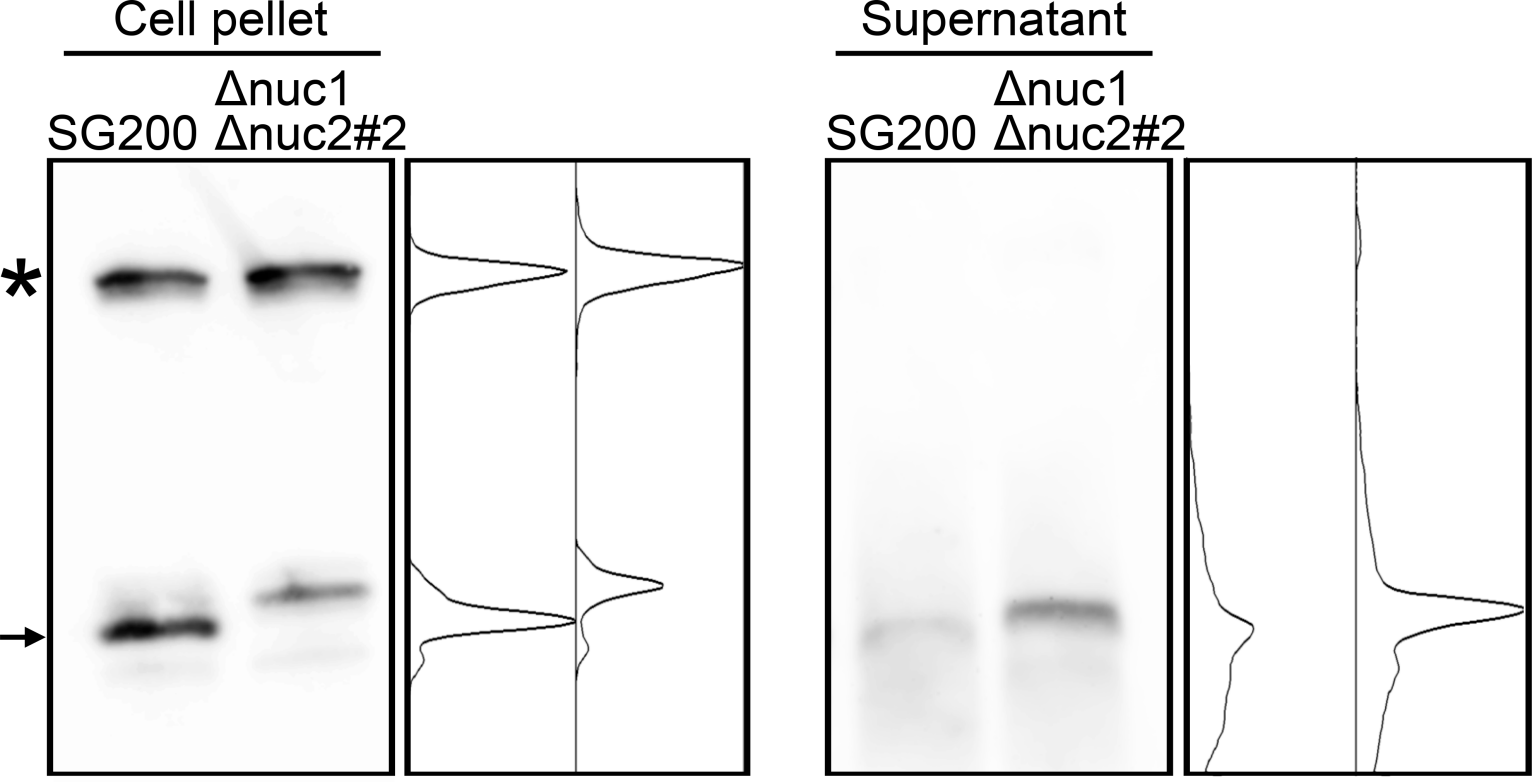
Northern blot analysis for the detection of tRNA-Gly fragment in culture supernatant of SG200 and SG200Δ*nuc1*Δ*nuc2* mutant. Asterisk indicates the full-length tRNA-Gly. Arrow indicates major 5’-tRNA fragment generated from full-length tRNA-Gly. Densitometry of tRNA fragment signals was shown at the right of respective panels.

Co-localization of Nuc1 and tRNA also imply that the tRFs would be secreted to extracellular space. To test this hypothesis, RNA was precipitated from culture supernatant of *U. maydis* strains and used for Northern blot analysis. In cell pellet fraction, full-length tRNA could be detected together with tRF (**Figure 6**). However, while full-length tRNA could not be detected at all in culture supernatant, tRF could be detected in culture supernatant (**Figure 6**). This result indicates that tRFs of *U. maydis* would be secreted to extracellular space.

## Discussion

In this study, we have identified the small RNAs in *U. maydis* that are derived from tRNAs and 5.8S ribosomal RNA by the cleavage with RNase T2. While *U. maydis* has lost its RNAi machinery during evolution, there have been no reports examining the small RNA fraction in fungal cells of *U. maydis*. This is the first report, to our knowledge, to investigate the small RNAs of *U. maydis*. Our small RNA-seq analysis demonstrated a number of tRNAs and 5.8S ribosomal RNAs are cleaved at either the anticodon loop of the host tRNA or stem loop of 5.8S ribosomal RNA. Furthermore, the population of those fragments are biased to 5’ end of certain tRNAs, suggesting the presence of specific machinery for the production of tRFs.

The molecular functions of the tRFs are presently unknown. One hypothesis is that the tRNAs may compete against potential RNase T2 substrates. In accordance with this notion, the activity of *Escherichia coli* RNase T2 (designated as RNase I) is known to be inhibited by helix 41 of 16S ribosomal RNA (Kitahara and Miyazaki, 2011). Conversely, in the model plant *A. thaliana*, RNase T2 family is necessary for ribosomal RNA decay (Hillwig et al., 2011). It would be interesting to examine the endogenous targets of RNase T2 using transcriptome analysis of RNase T2 knockout strains in *U. maydis*. Another possibility is that the degradation of tRNAs may alter intracellular tRNA repertoire, which may affect translational modulation and mRNA stability. In fact, our sequence data analysis suggests that the cleavage of tRNAs does not occur randomly, but rather occurs for specific tRNAs, which supports our hypothesis. Recently, methods for quantitative mature tRNA sequencing were developed (Pinkard et al., 2020; Nagai et al., 2021). This will shed the light to further understanding of tRNA repertoire in fungal cells of *U. maydis*. In mammalian tissues, tRFs have very long half-lives (~ days), which may be due to incorporation into RISC to form stable ribonucleoproteins (Shigematsu et al., 2014). Since tRFs in *U. maydis* cells also seem to accumulate stably, these fragments are likely to bind with RNA binding proteins. Therefore, the identification of proteinaceous factor(s) that bind to tRFs in *U. maydis* will be fascinating research project to demonstrate its function and the biological roles. In mammalian cells, Dicer or Angiogenin are known to be responsible for the production of tRFs (Yamasaki et al., 2009; Keam and Hutvagner, 2015; Liu et al., 2021). In contrast, RNase T2 family proteins are responsible for it in *S. cerevisiae* and *A. thaliana* (Thompson and Parker, 2009a; Megel et al., 2019), suggesting the diversity of biogenesis pathway for tRFs in respective organisms. However, the reason for such diversity in the mechanism of the cleavage of tRNAs remains unclear. Although the accumulation of tRFs are largely abolished in RNase T2 mutants of *U. maydis*, the mutants showed other faint signals after Northern blot analysis as shown in **Figure 3**. Therefore, there is likely to be other enzymes that are responsible for the production of tRFs in *U. maydis*.

Where the cleavage of tRNAs occurs is still under debating in other organisms (Thompson and Parker, 2009b; Keam and Hutvagner, 2015). In contrast, our study for the localization of Nuc1 and tRF (tRNA-Gly) suggested that the cleavage of tRNAs would occur during secretory processes in *U. maydis*. Supporting this, we could detect tRF in culture supernatant but full-length tRNA could not, indicating the secretion of tRFs to extracellular space. In rhizobia, it has been shown that the bacteria deliver tRFs to host legume plant to promote nodule formation (Ren et al., 2019). The rhizobial tRFs hijack the host RNAi machinery including Argonaute protein and regulate host genes that associate with nodule formation (Ren et al., 2019). In this context, *U. maydis* could also deliver tRFs to maize cells to hijack the RNAi machinery of host plants. However, the knockout strains did not show significant reduction of virulence in host maize. Similarly, the growth of RNase T2 knockout strains was not significantly different from that of wild type. Our results of the virulence contribution of RNase T2 genes in *U. maydis* is contradicting to the report by Mukherjee et al. (2020). Although we cannot formally rule out the possibility that RNase T2 genes have very minor impact on virulence in *U. maydis*, we consider that there are likely other biological significances of RNase T2 genes in *U. maydis*.

Our studies revealed the production and secretion of tRFs in *U. maydis*. However, the number of reports for the production of tRFs is still limited in plant filamentous pathogens (Nunes et al., 2011; Lambertz et al., 2015). We speculate that the production and secretion of tRFs might be a general molecular event in plant pathogenic fungi.

## Data availability statement

The datasets presented in this study can be found in online repositories. The names of the repository/repositories and accession number(s) can be found in the article/**Supplementary Material**.

## Author contributions

RY identified small non-coding RNAs, performed bioinformatic analysis and Northern blot analysis. FI and MY generated fungal strains and performed pathogenicity assay. ST performed western blot analysis, immunocytochemistry and *in situ* hybridization. RY and ST wrote manuscript with input from all co-authors. All authors contributed to the article and approved the submitted version.

## Funding

This work was supported by JSPS KAKENHI Grant Number 19K24688 for ST and 22K05565 for RY.

## Acknowledgments

We thank Masanori Kaido for his critical comments on the manuscript. We are also thankful to Regine Kahmann (Max Planck Institute for Terrestrial Microbiology, Marburg, Germany) for providing the seeds of *Zea mays* cv Golden Bantam.

## Conflict of interest

The authors declare that the research was conducted in the absence of any commercial or financial relationships that could be construed as a potential conflict of interest.

## Publisher’s note

All claims expressed in this article are solely those of the authors and do not necessarily represent those of their affiliated organizations, or those of the publisher, the editors and the reviewers. Any product that may be evaluated in this article, or claim that may be made by its manufacturer, is not guaranteed or endorsed by the publisher.
