# Supplementary material for: The production and secretion of tRNA-derived RNA fragments in the corn smut fungus Ustilago maydis
Source: Front Fungal Biol. 2022 Aug 4;3:958798. doi: 10.3389/ffunb.2022.958798 (PMC10512261; doi:10.3389/ffunb.2022.958798)
Supplement: Supplementary file 1 [file DataSheet_1.pdf]

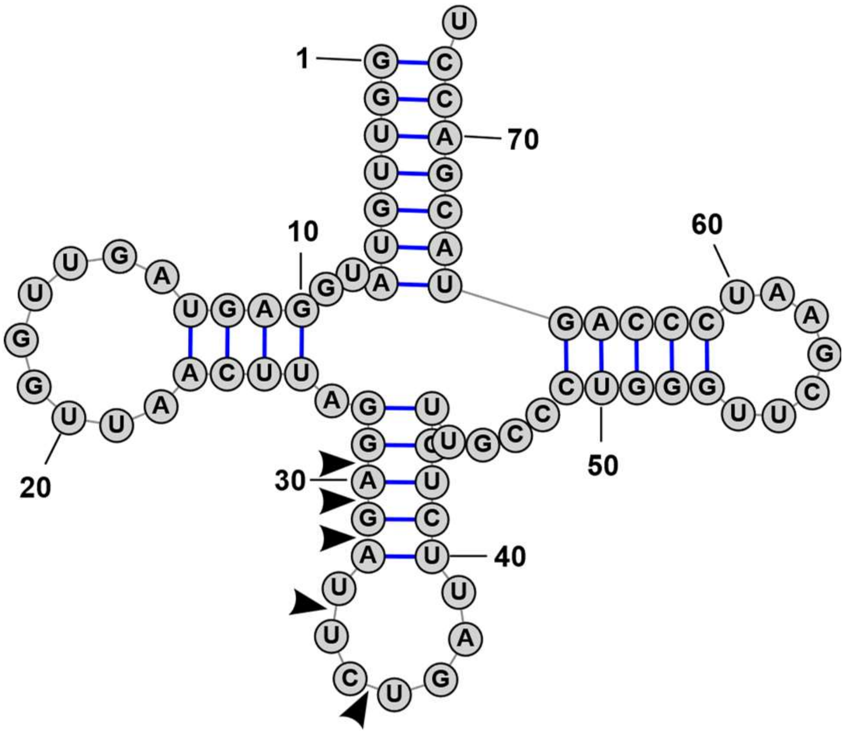

Supplementary Figure S1. Putative cleavage sites of tRNA-Gln *in U. maydis*.

Based on the sequence reads obtained by RNA-seq analysis, putative cleavage sites (black arrow heads) were predicted to tRNA-Gln (CTG-1-1). The numbers indicate the position of nucleotides from 5’ end of tRNA-Gln.

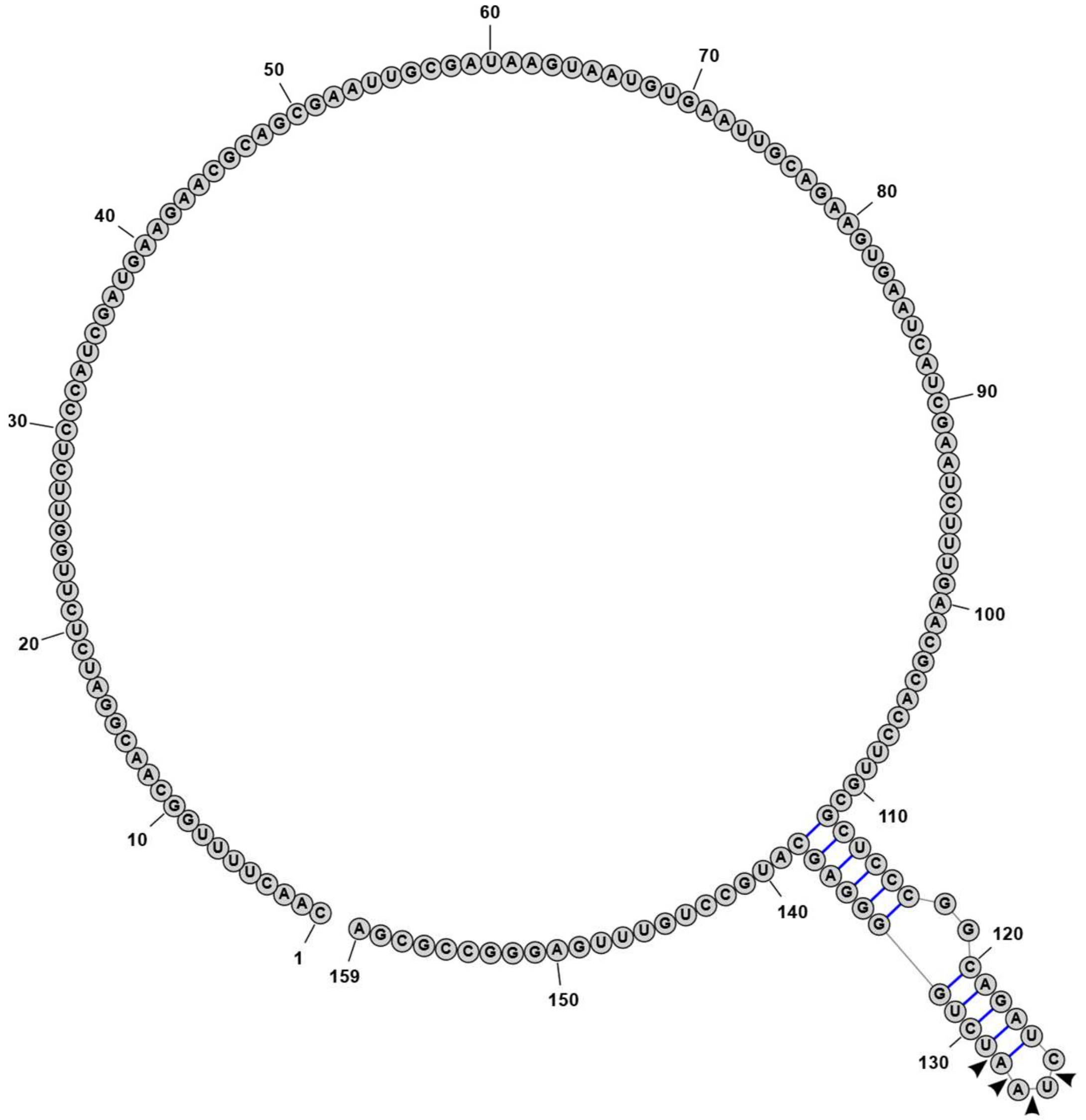

Supplementary Figure S2. Putative cleavage site of 5.8S ribosomal RNA in *U. maydis*.

Based on the sequence reads obtained by RNA-seq analysis, putative cleavage sites (black arrow heads) were predicted to 5.8S ribosomal RNA. The numbers indicate the position of nucleotides from 5’ end of 5.8S ribosomal RNA.

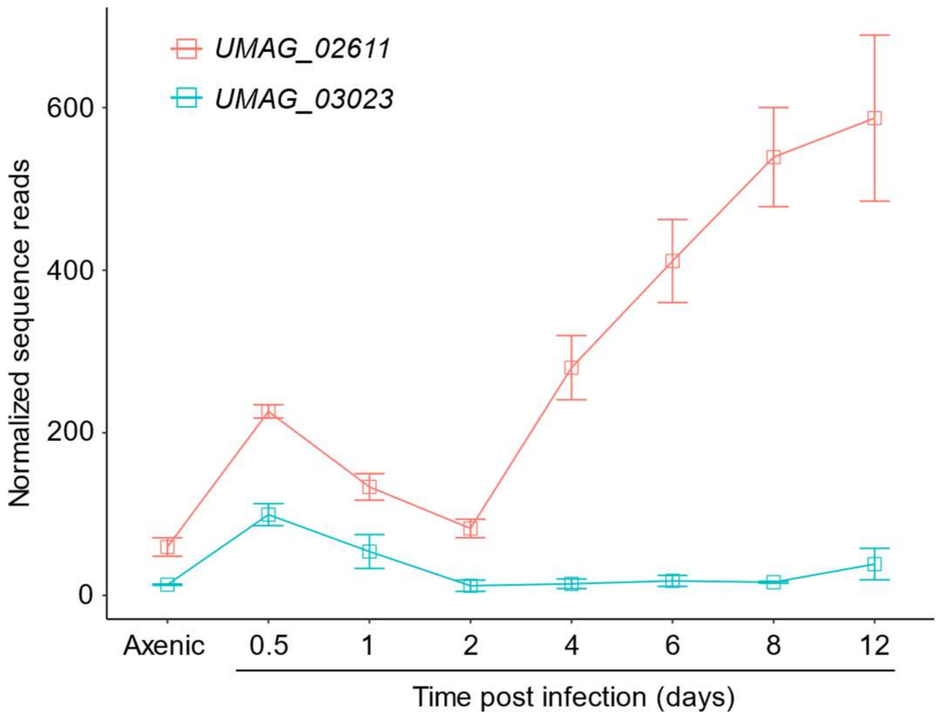

Supplementary Figure S3. The expression pattern of *nuc1* and *nuc2* genes during plant infection.

The normalized sequence reads retrieved from RNA-seq data (Lanver et al., 2018) were used to visualize expression pattern of *nuc1* (*UMAG_02611*) and *nuc2* (*UMAG_03023*) genes in axenic culture condition and at the time after plant infection. Error bars indicate standard deviation calculated from three biological replicates (Lanver et al., 2018) .

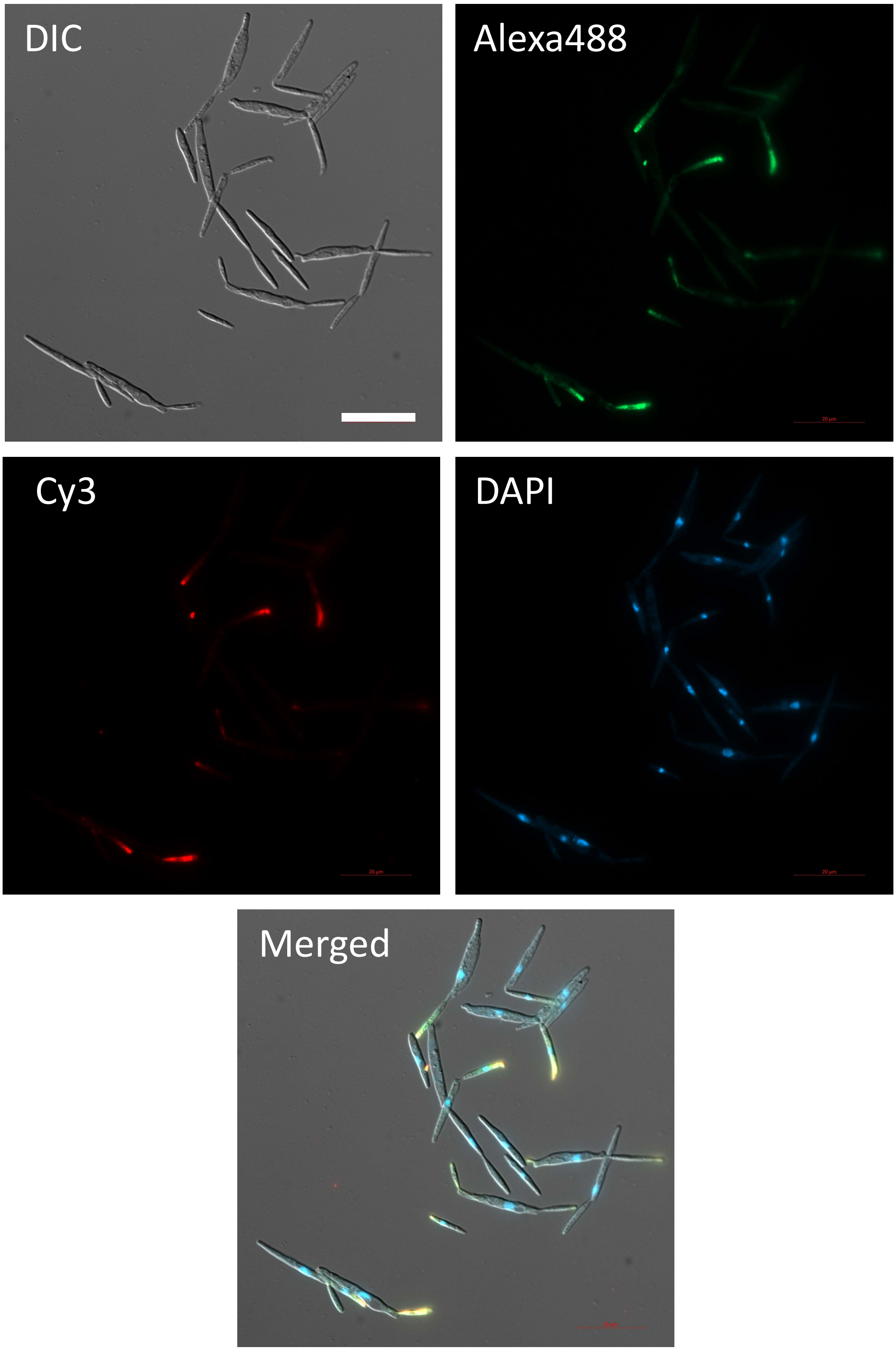

Supplementary Figure S4. Localization of Nuc1-HA and tRNA-Gly in sporidia cells of *U. maydis* strain expressing Nuc1-HA. Alexa488 fluorescence indicates Nuc1-HA. Cy3 indicates tRNA-Gly. DAPI indicates nuclei. Bar = 20 µm.
